# Fluorescence strategies for mapping cell membrane dynamics and structures

**DOI:** 10.1063/1.5143945

**Published:** 2020-05-12

**Authors:** Jagadish Sankaran, Thorsten Wohland

**Affiliations:** 1 Department of Biological Sciences, National University of Singapore, 117346 Singapore; 2 Centre for BioImaging Sciences, National University of Singapore, 117557 Singapore; 3 Department of Chemistry, National University of Singapore, 117346 Singapore

## Abstract

Fluorescence spectroscopy has been a cornerstone of research in membrane dynamics and organization. Technological advances in fluorescence spectroscopy went hand in hand with discovery of various physicochemical properties of membranes at nanometric spatial and microsecond timescales. In this perspective, we discuss the various challenges associated with quantification of physicochemical properties of membranes and how various modes of fluorescence spectroscopy have overcome these challenges to shed light on the structure and organization of membranes. Finally, we discuss newer measurement strategies and data analysis tools to investigate the structure, dynamics, and organization of membranes.

## INTRODUCTION

The cell membrane, made up of a diverse array of lipids, proteins, and carbohydrates, separates the cytoplasm from the external milieu and is arranged in a bilayer fashion consisting of an outer and an inner leaflet. The composition of the two leaflets of the cell membranes is different and is actively maintained by the cell. The physicochemical properties of the membrane are determined by the composition of the membrane. The physicochemical and functional properties of cell membranes have been investigated in two complementary ways so far, a top down approach by fluorescently labeling individual components in live cells and a bottom up reconstitution approach of sequential assembly of membranes by addition of individual lipids and proteins.

Lipids in aqueous solution self-assemble into micelles, lipid bilayers, and vesicles. Artificial lipid bilayers have been investigated at least since the eighteenth century,^1^ and since that time, we have gained a tremendous understanding of the organization^2^ and dynamics of artificial lipid bilayers. Phase diagrams for bilayer mixtures are widely available,^3,4^ and protocols to form vesicles of different compositions,^5–7^ with different domains^8^ even with leaflet asymmetry, have been published.^9^ A wide range of computational methods^10^ and non-destructive experimental techniques to probe bilayer dynamics have been developed. Despite our knowledge of artificial bilayers, the detailed intricate structure and dynamics of cellular plasma membranes remain unknown.^11,12^ A gamut of biophysical tools based on fluorescence spectroscopy,^13^ atomic force microscopy (AFM),^14^ nuclear magnetic resonance spectroscopy,^15^ and scattering^16^ have elucidated different aspects of the plasma membrane organization.

In this perspective, we discuss technical developments in fluorescence spectroscopy, which have provided new capabilities to measure, mine, manipulate, and model^17^ physicochemical properties of live cell membranes, and also how these techniques used in tandem might lead to further insights into the membrane structure and dynamics. This perspective is arranged in four parts. In the first part, we highlight the challenges in cell membrane investigations. In the second and the third part, we outline the various fluorescence techniques used to probe the in-plane diffusion, organization, cytoskeletal interactions, and the asymmetry of cell membranes. In the final part, we discuss how combinatorial microscopy and newer data analysis tools might lead to a better understanding of the structure and dynamics of the membrane.

## CHALLENGES IN CELL MEMBRANE INVESTIGATIONS

The structure and function of cell membranes are determined by the lipids and proteins constituting them. It is estimated that roughly a third of the prokaryotic genome codes for membrane proteins^18^ and at least 1000 different membrane lipid species have been identified.^19,20^ Lipidomic^20–23^ investigations have enabled us to map the compositional complexity of membranes across organisms, tissues, cell types, and organelles. In addition to the diversity of the membrane composition, the inner and outer leaflets of the bilayer have different compositions, and the coupling of the structure and dynamics between the two leaflets is still under intense investigation.

Apart from the complexities imposed by the intrinsic compositional heterogeneity in the study of cell membranes, the cell membrane is also affected by extrinsic factors such as interactions with structures in the vicinity. The cell membrane is influenced by adjacent ECM structures, including the cytoskeleton^24^ and the extracellular matrix, as well as neighboring and interacting cell membranes. Intercellular adhesion in tissues lead to the reshaping of cell membranes.^25^ The compositionally heterogeneous cell membrane associated with the structures in the vicinity is maintained in a state of dynamic equilibrium by a variety of active and passive processes, including exocytosis, endocytosis, and trans-bilayer crossing catalyzed by flippases and floppases. Passive processes in the cell membrane include in-plane diffusion and diffusion across the leaflets. Hence, in order to get a complete understanding of the cell membrane, it is important to not only study the lipids and proteins constituting the membrane but also the dynamic and structural matrices within which the cell membrane is embedded.

The membrane organization and the dynamics at the characteristic size of proteins on the scale of nanometers are vital for cellular transport, signaling, and function. Although membrane proteins constitute approximately only a quarter of the proteome, they constitute ∼60% of the drug targets.^26,27^ Hence, the physicochemical principles underlying lipid interactions are important to aid in rational drug design against novel targets.

The lipid–lipid interactions in the cell membrane along with membrane–cytoskeletal interactions lead to large scale visible domains^29–34^ with long lifetimes, which are easily imaged, or sub-microscopic domains with lifetimes in the millisecond range, which are difficult to capture.^35^ At least some of the structural determinants of the various proteins for raft associations have recently been determined.^36^ Although the domains might be small and highly dynamic, a large membrane fraction can be domain occupied at any point in time. It is therefore necessary to study the complex interplay between the membrane structure and dynamics at a variety of spatiotemporal scales to understand their impact on the membrane function. Functionally, compositional compartmentalization in membranes has been implicated in cell signalling,^37^ vesicle and protein trafficking, and pathogen entry and exit.^38^ Compartmentalization within a spatial domain leads to increased protein–protein interactions, leading to signal amplification. Since the compartmental structures are dynamic, their size and shape undergo changes in response to intracellular or extracellular stimuli.

The fragility of the dynamic state of the plasma membrane is demonstrated by the fact that it can be so easily destroyed. Artificial bilayers have not been able to reproduce plasma membrane dynamics and even giant plasma membrane vesicles (GPMVs) that are directly produced from live cells do not reconstitute the dynamics of cells as can be seen by the very different behavior of the same probe molecules in cells and GPMVs.^39^ This could partly be due to the inability of GPMVs to recapitulate dynamic processes such as exo- and endocytosis and the associations with the missing cytoskeleton and extracellular matrix. Hence, it is imperative to study the molecular dynamics (MD) of membrane constituents in their native state.

Given the heterogeneity in sizes and dynamics of domains in a cell membrane, it has been challenging to image these structures directly using optical microscopy since the sizes are below the resolution limit and the domains are putatively too short-lived to be imaged. Hence, a wide variety of fluorescence based techniques have been utilized to infer the physicochemical properties of domains in live cell membranes while also avoiding the artifacts of cell fixation.^40^

## TECHNIQUES USED IN THE STUDY OF MEMBRANES

The techniques described here are grouped into two main classes, those that are used to monitor the in-plane diffusion of molecules and those that are used to monitor the organization of the membrane. Fluorescence correlation spectroscopy (FCS), fluorescence recovery after photobleaching (FRAP), and single particle tracking (SPT) are three fluorescence based biophysical techniques used to monitor the in-plane diffusion of molecules. The main tools to investigate organization in a membrane are homo-Förster resonance energy transfer (homo-FRET), generalized polarization (GP), fluorescence lifetime imaging microscopy (FLIM), FCS diffusion laws, and brightness monitoring techniques. Apart from membrane organization, the oligomerization state of proteins embedded in membranes is determined by brightness monitoring techniques such as number and brightness analysis (N&B), photon counting histogram (PCH), and fluorescence intensity distribution analysis (FIDA).

The spatial and temporal scales probed by different techniques are listed in Table I. It has to be noted that there are two different relevant temporal scales for several of the techniques. There is an intrinsic resolution at which processes can be measured. For instance, in the case of FCS, this can be as low as ns and reach well above the second scale. Apart from the intrinsic temporal resolution of the technique, there is a total measurement time. Typically, for all the techniques, the total measurement time is in the scale of tens of seconds to a minute. Hence, all discussed techniques can detect changes in the dynamics of the sample being investigated in timescales of seconds to minutes.

**TABLE I.** Spatial and temporal length scales probed by different techniques. NA: not applicable.

Techniques	Reported membrane physicochemical property	Spatial scale	Intrinsic temporal scale^*^	References
FCS	Diffusion coefficient and concentration	∼200 nm	ns to s	43
FRAP	Diffusion coefficient and fraction of immobile particles	∼*μ*m	s to min	47
SPT	Mode of diffusion	∼10 nm	ms	48
Homo-FRET	Membrane organization	∼10 nm	ns	49
GP	Microenvironment of the probe molecule	∼10 nm	ns	50
FLIM	Microenvironment of the probe molecule	∼10 nm	ns	51
FCS diffusion law	Mode of diffusion	∼100 nm	ns to s	52
N&B	Concentration and oligomerization state	∼200 nm	NA	53
PCH	Concentration and oligomerization state	∼200 nm	NA	54

^*^Apart from the intrinsic temporal resolution of the technique, there is a total measurement time. The total measurement time for all the techniques in Table I is in the scale of tens of seconds to a minute.

### Fluorescence techniques to probe diffusion

Fluorescence correlation spectroscopy (FCS), fluorescence recovery after photobleaching (FRAP), and single particle tracking (SPT) are three fluorescence based biophysical techniques used to quantitate the diffusion coefficient of lipids diffusing in a membrane.^41^ FCS and FRAP are techniques with their theoretical underpinnings in the fluctuation dissipation theorem. SPT is a single molecule technique in which the diffusion coefficient is estimated by directly observing the movement of single molecules for a certain period of time.

If a system in thermal equilibrium is perturbed by an external force, then the system returns to equilibrium at a characteristic time depending on the process dissipating the fluctuation.^42^ Even without a perturbation, spontaneous thermal fluctuations are also dissipated at a characteristic time depending on the process dissipating the fluctuations. FCS^43–45^ takes advantage of these spontaneous fluctuations around equilibrium and quantifies the mobility of molecules by monitoring the timescale of inherent fluctuations in fluorescence of diffusing molecules at thermal equilibrium, whereas FRAP^46^ quantifies the mobility of diffusing fluorescent molecules by monitoring the time taken for the fluorescence to recover to thermal equilibrium after an induced perturbation. FCS provides a measure of sub-micrometer uniformity, while FRAP provides estimates at the micrometer scale. Using the exact same data of a sample, the two techniques were shown to provide the same results with FRAP typically having larger errors due to difficulties in finding appropriate photobleaching correction procedures and fit functions.^55^

### FRAP

**FIG. 1.**
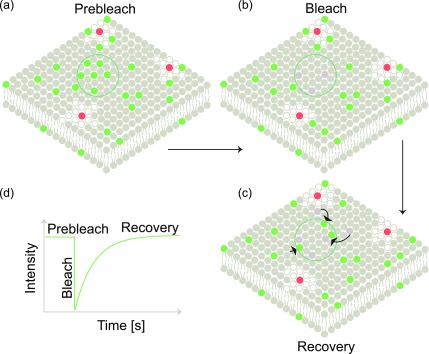
Fluorescence recovery after photobleaching: (a) a fractional area indicated by the green circle is photobleached leading to (b) depletion of fluorescent molecules in that area. (c) Fluorophores from the surrounding regions diffuse into the bleached area, leading to recovery of fluorescence in the marked green circular area. (d) Schematic intensity plot showing the pre-bleach intensity, bleaching, and recovery in the circular area.

In FRAP, a fractional area is photobleached, and the time taken for the fluorescence to recover is monitored (Fig. 1). The recovery can either be diffusion- or reaction-limited.^47^ If the diffusion is the slowest process, the recovery is diffusion-limited and the recovery time is related to the diffusion coefficient. In the case of a reaction-limited recovery, fast bulk diffusion is coupled with slow adsorption/desorption kinetics. In the case of reaction-limited processes, bleaching is performed at large areas such that surface diffusion does not affect the recovery.^56^ Depending on the experimental design, FRAP yields information about diffusion and/or absorption/desorption kinetics.^57^

### FCS

**FIG. 2.**
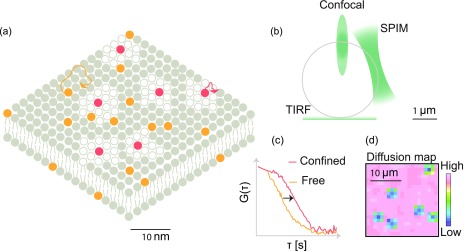
Fluorescence correlation spectroscopy: (a) a snapshot of the molecular organization of a compartmentalized lipid bilayer. Fluid phase regions are labeled with an orange fluorophore, while the domains are labeled with a red fluorophore. (b) In the case of a unilamellar vesicle, different bilayer regions are probed by the three configurations of FCS discussed in this perspective. The exponentially decaying evanescent wave in TIRF is used to investigate the region attached to the substrate. Confocal and SPIM based FCS are used to investigate the apical regions of the vesicle. (c) FCS measurement functions for slowly diffusing red fluorophores and rapidly diffusing orange fluorophores. The two functions differ in their temporal decay with the slowly diffusing fluorophore exhibiting the slower decay and wider FCS curve. (d) In imaging FCS in TIRF or light sheet mode, one obtains a diffusion map depicting regions of varying mobilities in the field of view of the membrane under investigation.

The diffusion coefficient is quantified in FCS (Fig. 2) by statistically analyzing the fluorescence fluctuations caused by single fluorescent molecules transiting through a small observation volume within a larger sample containing an ensemble of molecules at equilibrium. The fluorescence fluctuations carry information about the average residence time of the molecules in the small observation volume. The diffusion coefficient is then calculated from the average residence time, and is inversely proportional to the average residence time. Apart from diffusion coefficients, FCS determines the concentration of the transiting molecules.

FCS is mostly conducted in confocal setups measuring diffusion a single point at a time. However, FCS can also be performed in an imaging mode either using scanning FCS or by a total internal reflection fluorescence (TIRF) or a single plane illumination (SPIM) microscope, referred to as Imaging FCS^41,58–60^ [Fig. 2(b)].

Scanning and imaging FCS provide spatially resolved maps of diffusion coefficients [Fig. 2(d)]. Since these approaches measure membrane fluidity at many points simultaneously, they can be used as a probe for spatial uniformity^61^ of the membrane. Domains of differing mobilities interspersed in an otherwise region of uniform mobility leading to a mosaic pattern in the diffusion maps can be easily distinguished from uniform diffusion maps. The heterogeneity in diffusion on a membrane can be visualized by performing cross-correlations of points separated across space as performed in pair correlation function (PCF) analysis^62^ or ΔCCF distribution analysis.^63^ One of the advantages of Imaging FCS is that the illumination area can be varied post acquisition. Hence, a single experiment can yield insights into the diffusion of lipids in a membrane on the sub-micrometer to micrometer scale.

While confocal setups illuminate the whole sample, TIRF and SPIM based measurements illuminate only the parts of the sample, which are observed, allowing longer measurements with less photodamage. Another advantage in TIRF based Imaging FCS is that it avoids background noise due to the contributions from the bulk liquid away from the interface.

### SPT

**FIG. 3.**
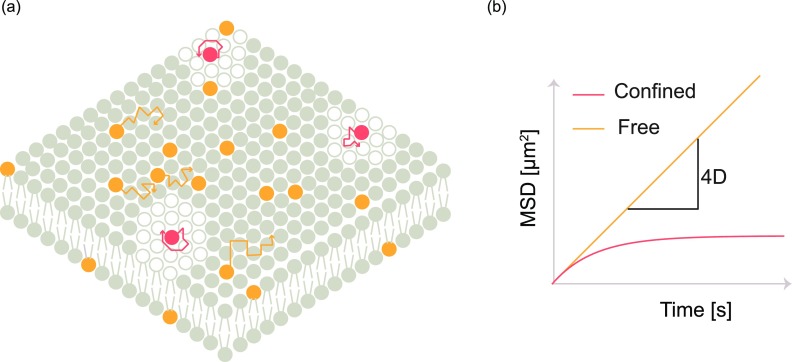
Single particle tracking: (a) orange fluorophores are used to label the freely diffusing fluid phase, while red fluorophores are confined in domains. Individual fluorophores are tracked as shown by orange and red trajectories. (b) The estimated mean squared displacement (MSD) at various time intervals is plotted and fitted to yield the mobility parameters.

In SPT, a labeled fluorophore is tracked over time to determine the mean squared displacement (MSD) during a certain time (Fig. 3), based on its trajectory.^48,64^ The obtained displacement over time is mathematically transformed to estimate the transport coefficient. SPT has been used to study a variety of transport phenomena such as free diffusion, directed flow, and anomalous diffusion.^65–67^ Within a single trajectory, there are instances where the molecule can change its mode of diffusion. Such differences in modes of diffusion can be detected by change point analysis algorithms as the particle changes its mode of transport from diffusion to flow.^68^

Apart from localization and tracking based estimation of MSD, which is computationally intensive, MSD can also be obtained from correlation techniques.^69–73^ The apparent mean squared displacement can be obtained by inverting the autocorrelation curves obtained from FCS. The inversion of autocorrelation curves provides quantitative information about a variety of diffusion modes, including free diffusion, continuous time random walk (CTRW), caged diffusion, obstructed diffusion, two-state diffusion, and diffusing diffusivity.^73^

### Fluorescence techniques to probe organization

The main tools to investigate organization in a membrane are homo-FRET, generalized polarization (GP), fluorescence lifetime imaging microscopy (FLIM), FCS diffusion laws, and brightness monitoring techniques. Apart from membrane organization, the oligomerization state of proteins embedded in membranes is determined by brightness monitoring techniques such as number and brightness analysis (N&B), photon counting histogram (PCH), and fluorescence intensity distribution analysis (FIDA).

### Homo-FRET

Energy transfer techniques used in the study of membranes are FRET and homologous-FRET (homo-FRET). This group of techniques is based on the fact that there is a non-radiative energy transfer from a donor fluorophore to an acceptor fluorophore located within ∼10 nm of each other. The efficiency of energy transfer between the donor and the acceptor serves as a readout of the distance between the molecules. In the case of FRET, the emission spectrum of the donor has a significant overlap with the excitation spectra of the acceptor. In the case of homo-FRET, there is a significant overlap of the excitation spectrum with the emission spectrum of the same molecule.

**FIG. 4.**
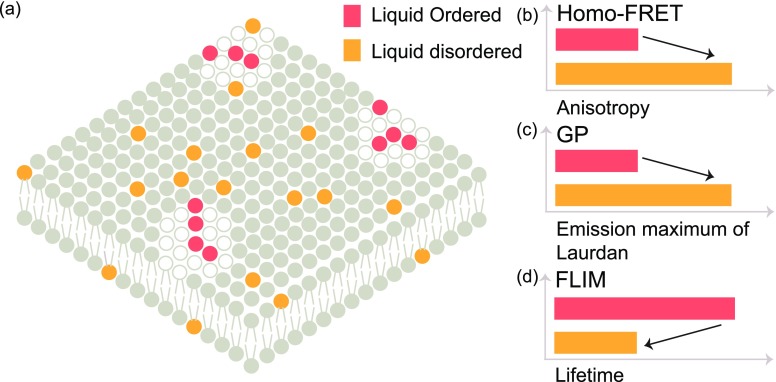
Techniques to probe organization: (a) snapshot of a compartmentalized membrane where the red fluorophores are localized in liquid ordered domains, while the orange fluorophores are localized in the liquid disordered fluid phase. (b) Homo-FRET, (c) GP, and (d) FLIM are techniques used to study organization in membranes. The lifetime of the fluorophores as quantified in FLIM and the anisotropy as quantified by homo-FRET are used to investigate local microenvironments. In general, polarization microscopy monitors the environmentally sensitive shift in the emission maximum of laurdan to determine the local membrane structure.

Typically, the intensity of fluorescence emission is anisotropic along different axes of polarization. The fluorescence emission has a similar polarization to that of the incident light except the depolarization caused due to particle rotation and the differences between the absorption and the emission dipole orientation. Homo-FRET occurs between molecules with various dipole orientations causing further depolarization, leading to a reduction in fluorescence anisotropy.^49^ Hence, fluorescence anisotropy measured by quantifying the depolarization caused by the sample upon excitation with polarized light serves as a readout for homo-FRET. The anisotropy of the region under investigation depends on the viscosity of the region and the molecular organization in that area [Fig. 4(b)].

### GP

The microenvironment of a bilayer can also be probed by the use of polarity sensitive probes such as laurdan,^74–77^ which reports on the fluidity and order of the bilayer. Generalized polarization [Fig. 4(c)] is a spectroscopic property exhibited by polarity sensitive probes that exhibit a shift in emission maximum depending on the local organization of the lipid phase.^50^ Recently, laurdan based imaging has also been demonstrated in a whole organism.^78^

### FLIM

Apart from general polarization, the microenvironment in the membrane can also be investigated by quantifying the fluorescence lifetime.^51^ The fluorescence lifetime is a molecular property independent of the fluorophore concentration providing an estimate of the average time spent by the molecule in the excited state. The fluorescence lifetime depends on the viscosity of the local environment. An increase in viscosity leads to an increase in the fluorescence lifetime [Fig. 4(d)]. In the case of FLIM, spatially resolved measurements of lifetimes are performed. FLIM performed using molecular rotors enabled the quantification of microscopic viscosity of liquid ordered and liquid disordered phases in lipid membranes.^28^

### FCS diffusion laws

**FIG. 5.**
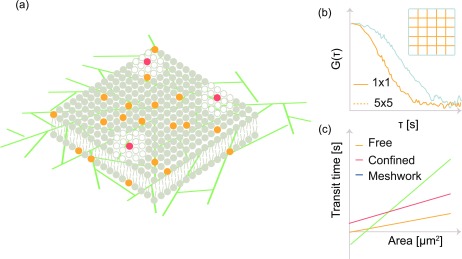
FCS diffusion laws: (a) a compartmentalized cell membrane along with the underlying cytoskeleton. In the FCS diffusion law, the diffusion time at increasing observation areas is determined by fitting the respective autocorrelation functions. (b) The autocorrelation function for a small bin size decays rapidly when compared to an autocorrelation obtained from a larger area. (c) The transit time vs area is fitted using a linear function. The intercept value serves as the metric to distinguish between free diffusion, confined diffusion, and diffusion within a mesh.

FCS diffusion laws are used to investigate organization in membranes [Fig. 5(a)]. The diffusion coefficient, measured in area per unit time (m^2^/s), for free diffusion is independent of the area over which it is observed. Therefore, the average residence time of molecules is directly linear proportional to the observed area. Representative autocorrelation functions at two different bin sizes are shown in Fig. 5(b). Experimentally, plots of the average residence time vs the observed area, referred to as diffusion laws, provide a linear plot with a zero y-intercept [Fig. 5(c)]. Any deviations from linearity or non-zero y-intercepts are an indication of inhomogeneity in diffusion within the observation area. Non-linearity in such plots^52^ is typically an effect of domain or hop diffusion due to hindrance by the cytoskeleton. The diffusion laws were first used in FRAP^77^ and later extended to FCS.^52^

The diffusion law plot is analogous to the MSD plot in SPT. In the case of SPT, MSD is plotted vs time where the slope is directly proportional to the diffusion coefficient. In the case of the FCS diffusion law, the average residence time at various areas is plotted against the area. The slope of the plot is inversely proportional to the diffusion coefficient.

FCS diffusion law studies showed that glycosyl phosphatidylinositol (GPI) anchored proteins preferentially partition into lipid domains.^80^ Sterol and sphingolipid rich lipid domains of size between 10-200 nm are referred to as lipid rafts.^29^ Apart from localization, FCS diffusion law experiments have shed light on how raft localization has an influence on signaling and hence on the function of the biomolecule. Performing the diffusion law by varying the size of the illumination spot is referred to as spot-variation FCS.^81^ An alternative approach is the use of the z-scan^82^ where FCS is performed at various heights below and above the plane of the membrane.

Imaging FCS has the advantage that it simultaneously records multiple contiguous pixels that can be combined to provide the various observation areas needed for an FCS diffusion law analysis. Imaging FCS thus provides better statistics as it records multiple pixels simultaneously, and it calculates the diffusion law from a single measurement. By contrast, multiple measurements per observation area are necessary for point FCS based computation of the diffusion law leading, to longer measurement times and possible artifacts caused by photodamage due to repeated excitation.

### Brightness techniques

Each fluorescent molecule has an inherent brightness depending on the structure of its fluorophore. The inherent brightness depends on the extinction coefficient and the quantum yield of the molecule. Oligomerization of fluorescent molecules leads to an increase in the brightness. Hence, quantifying the brightness of a fluorescent molecule provides insights into its aggregation state. The brightness of a molecule can be estimated by performing correlation spectroscopy either in the spatial or in the temporal domain. Estimation of brightness by computing the spatial autocorrelation function is referred to as image correlation spectroscopy (ICS).^83,84^ ICS has been employed to study the spatial distribution of membrane receptors.

**FIG. 6.**
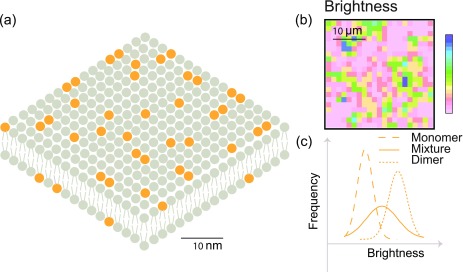
N&B analysis: (a) bilayer with orange fluorophores that exist as monomers or dimers. (b) The brightness is determined in each pixel and shown as a map. (c) Upon plotting the brightness values as a histogram and comparison with measurements for pure monomers and pure dimers, one can differentiate monomers, dimers, and a mixture of monomers and dimers.

In the case of temporal analysis, brightness is quantified in number and brightness analysis (N&B), photon counting histogram (PCH), and fluorescence intensity distribution analysis (FIDA). The mean value of a time varying fluorescence signal is the product of the number of particles in the observation volume and the average brightness of individual particles. The variance of the time varying signal is the product of the number of particles in the observation volume and the square of the average brightness of individual particles. Hence, the brightness of particles can be quantified as the ratio of the variance of a time varying fluorescence signal to its mean. This way of quantitation is referred to as number and brightness analysis^53,85^ (Fig. 6). N&B analysis has shed light on the oligomerization state of a variety of membrane proteins.

Apart from N&B and autocorrelation-based approaches, time varying fluorescence traces are analyzed by computing photon counting histograms (PCHs)^54^ or fluorescence intensity distribution analysis (FIDA)^86^ to estimate the brightness of molecules. Unlike the case of N&B, where the first two central moments of the distribution are used to estimate the brightness, theoretical models are fitted to the entire distribution of photon counts to estimate the brightness and the average number of particles in PCH and FIDA.

## MEMBRANE ASSOCIATED PHENOMENA PROBED BY FLUORESCENCE TECHNIQUES

In this section, we describe how the different techniques described above have enabled us to quantify a wide variety of physicochemical properties of the membrane. The physicochemical properties described in this perspective are diffusion, organization, cytoskeletal interactions, and membrane asymmetry.

### Diffusion

FCS,^87^ FRAP, and SPT are used to quantify the diffusion coefficient on a membrane. The diffusion coefficient has been used as a proxy to quantify the roughness^88^ of the surfaces on which lipid bilayers were grown and as a measure of organization in the bilayer. Bilayers grown on rougher surfaces exhibit a lower mobility when compared to smoother surfaces. In the case of phase separated bilayers^89^ and cell membranes,^44^ the liquid disordered phase exhibits a larger mobility when compared to the liquid ordered phase.

### Organization

Apart from the self-organization of cellular membranes leading to compartmentalization, organization also refers to the oligomeric state of the individual lipid and protein components on the membrane. Oligomerization states are typically observed using FRET, PCH, and N&B analysis. The interested reader is referred here for a detailed review on oligomerization studies using FRET.^90^ PCH studies on the GPI-anchored urokinase plasminogen activator receptor^91^ yielded insights into the protein's dynamics and aggregation state. The cluster sizes can also be estimated from the pair correlation computed in the spatial domain. The pair correlation based approach has yielded insights into the organization of GPI anchored proteins^92^ and IgE-FcεRI.^93^ Apart from FRET and PCH, oligomerization of a wide variety of molecules has been determined by N&B.^94,95^

The organization in a membrane has been observed by homo-FRET, FRAP, SPT, and FCS diffusion laws. Homo-FRET measurements^96,97^ have enabled the quantification of cluster sizes of GPI anchored proteins and hedgehog proteins.^98^ Homo-FRET has also been utilized to delineate the role of activity of cortical actin^99^ and integrin mechano-chemical signaling^100^ in the formation of the GPI-anchored clusters.

FRAP experiments revealed that there is dynamic partitioning of molecules in and out of raft regions.^101^ SPT experiments enabled the visualization of non-Brownian diffusion phenomena called hop diffusion^102^ where molecules jumped across diffusion barriers in the cell membrane. SPT studies showed that raft associated proteins exhibited a slow diffusing regime corresponding to diffusion in raft associated areas and a fast diffusing regime while diffusing in the homogeneous fluid phase.^103^

The following studies are examples of the use of FCS diffusion laws to elucidate the membrane organization on live cells. Akt signaling gets facilitated by the localization of Akt proteins into nanodomains upon PIP3 accumulation in the membrane.^104^ Domain confinement of activating receptors led to tolerance in Natural Killer (NK) cells.^105^ A combination of super-resolution with FCS diffusion laws showed that GPI anchored proteins are trapped in 20 nm domains in a live cell.^35^ Imaging FCS studies have been utilized to understand the dynamics of epidermal growth factor receptors (EGFRs)^106^ in cells and Wnt3 localization in rafts^107,108^ even in organisms. The organization of the plasma membrane has already been shown to be dependent on the cell line used for investigation.^109^ The abundance of ceramides in different cell lines has been shown to play a role in altering membrane dynamics and organization.^110^ A comparison of organization of the membrane from a cell line with the organization of the membrane inside the tissue of a live organization will enable one to delineate the importance of the 3D microenvironment in shaping the arrangement of the membrane inside a live organism.

### Interactions with the cytoskeleton

The cytoskeleton is known to influence the diffusion of molecules in the lipid membrane. As discussed in the section on FCS, one of the ways of monitoring the association is through the use of the FCS diffusion law. It has been shown that a fusion protein consisting of a plasma membrane targeting domain of X-linked retinitis pigmentosa protein RP2 with a green fluorescent protein (GFP) preferentially associates with the cytoskeleton,^111^ and EGFR dynamics are influenced by the cytoskeleton. More recently, the FCS diffusion law has been extended to analyze complex diffusive modes where the biomolecule under investigation is influenced not only by domain confinement but also influenced by the cytoskeleton.^112^ Such an approach will enable one to effectively decouple the relative influences of the cytoskeleton and domain organization on the diffusion of molecules in the cell membrane.

### Asymmetry and coupling

A variety of protocols based upon enzymatic activity, microfluidics, and vesicle fusion are now available to create asymmetric lipid bilayers.^113^ Biophysical investigations using such asymmetric lipid bilayers have enabled us to understand the coupling between the two leaflets and to understand the role played by asymmetry and coupling in nanodomain formation.^114^ Membrane asymmetry studies are performed with probes, which specifically target only to a certain leaflet of the membrane.

FCS experiments using asymmetric model membrane vesicles demonstrated that acyl chain interdigitation caused by lipids in an ordered outer membrane leaflet led to a reduction in the lateral diffusion of inner membrane lipids, whereas FLIM experiments demonstrated that membrane order was decoupled between the inner and the outer leaflet.^115^ A combination of FLIM and Imaging FCS^116^ demonstrated that the inner leaflet of the cell membrane existed in a liquid disordered phase, and the molecules exhibited free diffusion in the inner leaflet. Cell line specific differences in membrane asymmetry^116^ have been reported, and the molecular details leading to cell line specific behavior remain to be elucidated.

## CHOICE OF FLUORESCENCE TECHNIQUES TO STUDY MEMBRANE PROPERTIES

**FIG. 7.**
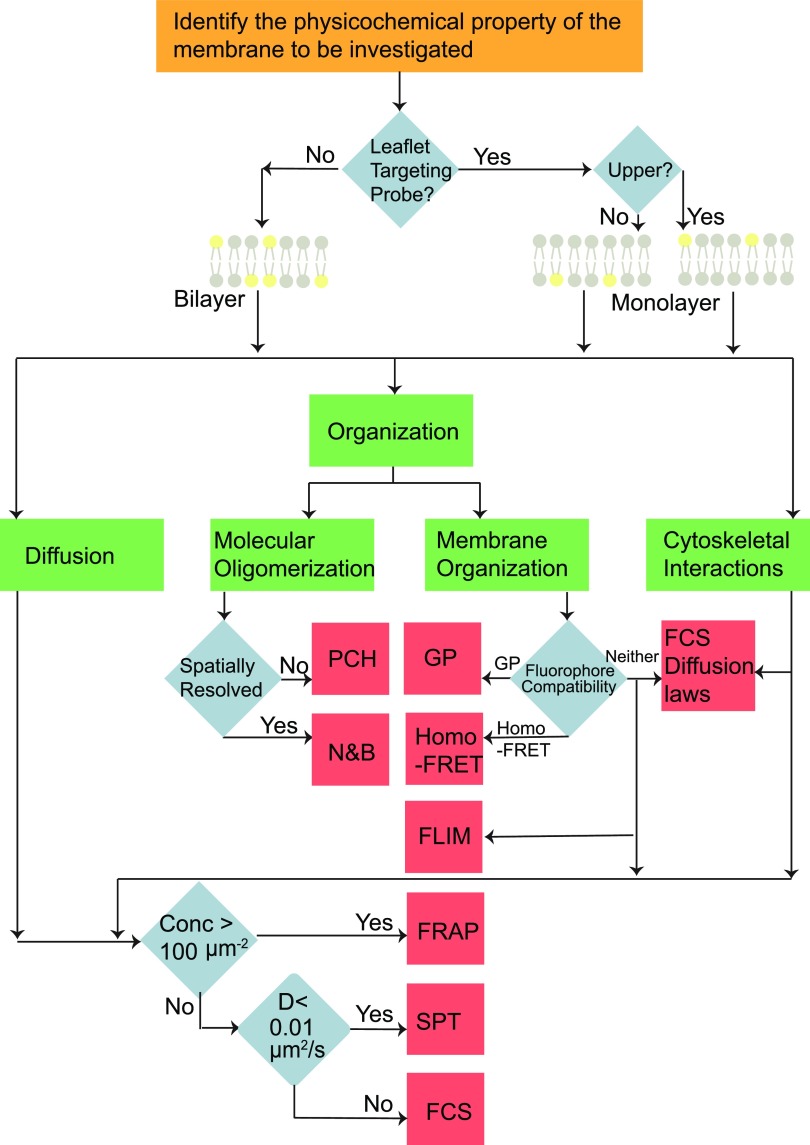
Identification of suitable choice of fluorescence technique: a flow chart to guide the choice of technique depending on the membrane property being investigated. Diffusion: diffusive motion can be measured with FCS,^41^ FRAP,^41,117^ and SPT.^118^ FCS is insensitive to immobile particles and less sensitive to slow moving particles with a diffusion coefficient <0.01 *μ*m^2^/s.^118^ FRAP can be performed at concentrations as high as 100 *μ*m^−2^, which is a concentration range inaccessible to FCS or SPT. Molecular oligomerization: PCH is suitable for single point measurements on a cell to determine the oligomerization state of biomolecules, whereas N&B is suitable for obtaining spatially resolved measurements of the oligomeric state of biomolecules. Membrane organization: two techniques that are typically used to investigate membrane organization are GP and homo-FRET. The excitation and emission spectra of the fluorophore must have considerable overlap in order to perform homo-FRET. GP is typically performed with fluorophores that exhibit a change in emission maximum depending on the local microenvironment. Apart from GP and homo-FRET, membrane organization can also be studied using FRAP,^101^ FCS,^44^ and SPT.^103^ Cytoskeletal interactions: the interaction of biomolecules with the cytoskeleton can be investigated using diffusion monitoring techniques (FCS, FRAP, and SPT). Interactions with the cytoskeleton have been investigated indirectly using diffusion laws, which were first demonstrated in FRAP^79^ and then in FCS.^52^ SPT measures this interaction directly by probing the movement of single molecules in relation to the cytoskeleton. Asymmetry and coupling: membrane asymmetry studies are performed with probes that specifically target only a certain leaflet of the membrane. With such targeted probes, the full range of techniques can be used to study the various properties of the single leaflets.

There are multiple techniques available to probe membrane physicochemical properties. The flow chart shown in Fig. 7 will aid in choosing a suitable technique depending on the membrane property being investigated. Apart from the membrane property, the choice of the technique depends on the properties of the sample being investigated. In the case where the chosen fluorophore is a specific leaflet targeting probe, the physicochemical properties of only the particular leaflet are investigated, whereas in the case where the fluorophore partitions equally in both the leaflets, the physicochemical properties of the bilayer is investigated using the techniques.

Diffusive motion is investigated with FCS,^41^ FRAP,^41,117^ and SPT.^118^ Although the three techniques have overlapping concentration and diffusion coefficient ranges in which they are applicable, each one has an exclusive range where it performs better than the other techniques. FCS is insensitive to immobile particles and less sensitive to slow moving particles with a diffusion coefficient <0.01 *μ*m^2^/s.^118^ Although FRAP cannot be performed at very low concentrations of fluorophores, FRAP can be performed at concentrations as high as 100 *μ*m^−2^, which is a concentration range inaccessible to single molecule sensitive techniques. The choice between PCH and N&B to determine the oligomerization state is based on the need to perform single point measurements (PCH) vs spatially resolved measurements (N&B). The choice between GP and homo-FRET to investigate membrane organization depends on the fluorophore used to label the membranes. The excitation and emission spectra of the fluorophore must overlap in order to perform homo-FRET. GP is typically performed with fluorophores, which exhibit a change in emission maximum depending on the local microenvironment.

## HYPHENATED METHODS OR TANDEM METHODS

Typically, the contrast in fluorescence imaging is provided by time-averaged intensities. Parametric maps from fluorescence spectroscopy utilizing mobility, lifetime, anisotropy, or energy transfer efficiency as contrast parameters have enabled the visualization of the physicochemical state of molecules. Apart from fluorescence based techniques, electrical and scanning based techniques are used in the investigation of the membrane structure. Electrical methods of detection are typically used to report on the integrity and continuity of the membrane on the surface. Scanning probe microscopy is utilized to quantify the roughness of the surface. In the case of densely packed proteins with an almost static structure, the details of organization can be gained by atomic force microscopy (AFM).^119^ Apart from organization, conformational dynamics of transmembrane channels in lipid patches has been monitored by high-speed AFM performed at rates of ∼10 frames per second.^120^

There have been many hyphenated techniques combining optical microscopy with scanning probe microscopy and impedance spectroscopy not only to probe multiple biophysical properties at the same time enabling quantification but also to obtain even correlations between different parameters at the same time. Hyphenated techniques such as TIRF-AFM,^121–124^ AFM-FCS,^125^ FCS-anisotropy,^126^ and near field scanning optical microscopy (NSOM-FCS)^127^ have been demonstrated so far.

A combination of AFM with fluorescence microscopy allows correlating topographical and fluorescence images. A study^128^ utilizing a combination of AFM and fluorescence microscopy highlighted that great caution must be exercised when concluding about the preferential localization of membrane probes from fluorescence microscopy. The authors showed that many probes associated with lipid rafts preferentially partitioned into non-raft regions in AFM images. This study showed that the coupling of a fluorophore to a lipid leads to an abolishment of its ability to partition into raft localized regions. Coupling an AFM to a polarized TIRF microscope^129–131^ enables one to obtain not only correlated fluorescence and topographical maps but also parametric images of the local order^132^ in the membrane.

As stated earlier, fluorescence anisotropy is dependent upon the rotation of molecules. As rotational diffusion, i.e., the orientational diffusion of the absorption and emission dipoles around the molecular axis (∼10–100 ns), happens on a much faster timescale than translational diffusion, i.e., the transit of the molecule through an optical diffraction limited observation area (∼10–100 ms), anisotropy tests viscosity on a much smaller scale than translational diffusion. To give an example, a molecule with a diffusion coefficient of 1 *μ*m^2^/s diffuses 200 nm in 10 ms, while the same molecule diffuses only 0.6 nm during a characteristic rotational diffusion time of 100 ns.

The use of NSOM for illumination in FCS led to a reduction in the observation area allowing the monitoring of organization of lipid membranes at sub-diffraction length scales. Apart from optical and surface tools, FCS has also been combined with another super-resolution microscopy technique called Super-resolution Optical Fluctuation Imaging (SOFI),^133^ which led to an improvement in the imaging resolution by a factor of 2.

It is important to note that in the case of hyphenated tools, the substrate must be conducive to both techniques. For instance, surfaces must be atomically flat, optically transparent, and conductive for atomic force microscopy, light microscopy, and electrical measurements, respectively. By adopting a modular design and by combining instrumentation from different techniques, it will be possible to simultaneously estimate the mobility, concentration, heterogeneity, surface roughness, conductivity, and order parameters from a single sample.

## COMPUTATIONAL METHODS IN MEMBRANE DYNAMICS

Today, an extensive amount of spectroscopic and imaging data can be generated in a very short time. New analytic tools are being developed to mine the data collected to obtain information about membrane dynamics. Bayesian^134,135^ approaches along with machine^136^ and deep learning^137,138^ have now been utilized to classify the modes of diffusion in single particle tracking. Apart from classification in SPT, a Bayesian framework^139^ is now available to analyze FCS data with shorter time traces when compared to conventional FCS analysis methods.

User autonomous data analysis paradigms will immensely aid the study of membrane dynamics by removing the human induced bias in data analysis. Two scenarios can be envisioned where machine learning will aid in data analysis. A network trained to be a classifier will assist the experimentalist in ascertaining the number of types of diffusion particles or whether other fluctuation processes such as photophysics or sample drift are involved. This will assist researchers in restricting the number of fitting models in FCS, FRAP, SPT, or FLIM. The most appropriate fitting model can then be determined by performing a Bayesian model selection^140^ or by systematically comparing experimental data to synthetic data obtained from numerical simulations.^141,142^

In the case of estimation of mobility, a trained regression network might estimate the diffusion coefficient and the concentration from the image stack itself eliminating the computational steps of calculating the autocorrelation functions in FCS or localization and estimation of mean squared displacement in SPT and fitting the function with theoretical models. Such data driven deep learning approaches might lead to an improved resolution of multiple diffusing particles better than that offered by current conventional curve-fitting approaches performed today.^143^

Apart from autonomous data analysis, deep learning approaches also hold great promise in the field of imaging membrane structure. Deep learning approaches have led to the transformation of diffraction limited images to super-resolved images.^144^ Such transformation techniques will lead to obtaining improved resolution of the membrane structure at sub-diffraction scales without the use of customized or specialized instrumentation. Apart from transformation, deep learning based image restoration^145^ methods also hold great promise in the field of membrane structure elucidation.

Advances in microscopy have provided an extensive wealth of information about the structural dynamics of the membrane. Such experimental developments have gone hand in hand with developments in the field of computational simulations of the membrane. Today's computational methods have the ability to probe membrane dynamics at atomic resolution and are referred to as “computational microscopes.”^146,147^ The increase in computational power over the last few decades has enabled the molecular dynamic (MD) simulations of even multi-component bilayer systems. The power at which the technology is developing, it is plausible that even the molecular dynamic simulation of an entire cell membrane might be possible in the next decade.^148^ The interested reader is referred here for a detailed discussion of the current state of the art simulation strategies to investigate cell membranes.^148^

## CONCLUDING REMARKS, AND FUTURE PROSPECTS

Fluorescence techniques that allow determining membrane dynamics and membrane nanoscale organization, as discussed in this review, are very important tools that helped shape our view of cell membranes as it is today. Based on the results from fluorescence microscopy and many other techniques, the picture of cell membranes emerging is one of a highly heterogeneous mixture of lipids and peripheral and membrane anchored proteins that transiently interact to form highly dynamic domains. The physicochemical principles underlying the formation of dynamic domains in model membranes are now well understood. Domains in model membranes have been directly imaged using fluorescence microscopy and other biophysical techniques. However, compartmentalization in cell membranes has always been inferred through a wide range of indirect biophysical measurements. Direct visualization of lipid rafts in living cells remains a challenge even today. Apart from the lack of direct visualization tools, the inherent spatiotemporal and compositional complexities in cell membranes pose significant challenges in the investigation of self-organization in cell membranes.

The cell membrane is influenced by static protein structures (cytoskeleton^149^ and extracellular matrix^150,151^) that can not only act as a base for membrane anchoring but also couple to lipids and proteins within the membrane and provide condensation nuclei or anchors for different biomolecules and domains. This system exists in a dynamic equilibrium that can quickly change and is driven by exocytosis and endocytosis events that can change the membrane composition and orchestrates the various membrane functions. Since the cell membrane, cytoskeleton, and ECM are dynamic, this leads to a complex dynamic membrane organization on multiple temporal and spatial scales, raising the question whether membrane organization and dynamics can be disentangled. Hence, approaches that can measure membrane organization at high spatiotemporal resolution are necessary. High spatiotemporal resolution implies also limited signals, which in turn requires an improvement of our data analysis methods to optimize information extraction from acquired data.

A combinatorial approach^152^ of mass spectrometry and lipidomics to decipher the bulk or spatially resolved^153^ composition of plasma membranes, super-resolution techniques to resolve the structure of protein complexes and structures within and adjacent to the plasma membrane, and novel single molecule sensitive spectroscopy methods to quantify the dynamics and interactions promises to resolve the highly dynamic structure of the plasma membrane leaflets. Ultrastructure investigations by electron microscopy (EM) in general and correlative light-electron microscopy (CLEM)^154,155^ and the new liquid phase EM^156^ in particular will be valuable in imaging membranes in the native state.

Molecular dynamics simulations are routinely performed at the millisecond timescale,^157^ which is the timescale of diffusion of lipids in membranes, and thus aid in deconstructing the heterogeneity associated with diffusion at different length and timescales observed in cell membranes using a bottom-up approach by systematically increasing the physicochemical complexity at every step. Combinatorial microscopy simultaneously performed with on-stage biological assays will allow obtaining correlations between the biophysical structure and function. Finally, all techniques discussed above are data intensive, and hence, parallelization using graphics processing units (GPUs) will lead to improvement in processing times.^158^ Newer data treatment strategies derived from machine learning will lead to user autonomous data analysis methods, thus removing the human induced bias in decision making. Machine learning based data treatment strategies, which incorporate knowledge from previously performed experiments used for training, also have the potential to improve the resolution of the estimated parameters.

Developments in combinatorial and correlated microscopy to image the structure, mass spectrometry to analyze the composition,^110,153,159^ and time-resolved spectroscopy to monitor the dynamics, assisted by advanced computation and molecular dynamics simulations,^148^ provide novel tools to address the challenges in the field of membrane structural dynamics and function and raise the hope of a more fundamental understanding of one of the arguably most intriguing cell structures, the cellular plasma membrane.
